# The Impact of the Interactivity of Internet Celebrity Anchors on Consumers’ Purchase Intention

**DOI:** 10.3389/fpsyg.2021.757059

**Published:** 2021-10-27

**Authors:** Weiguo Sun, Wei Gao, Ruoshi Geng

**Affiliations:** ^1^Business School, Changshu Institute of Technology, Changshu, China; ^2^School of Economics and Management, China University of Mining and Technology, Xuzhou, China; ^3^School of Management, Shanghai University, Shanghai, China

**Keywords:** internet celebrities, interactivity, social presence, flow experience, livestreaming marketing

## Abstract

The current study focuses on a novel and recently popular internet phenomenon – celebrity livestreaming marketing. As one of the primary advantages of livestreaming marketing by Internet celebrities, we propose that the timely interactivity of Internet celebrities plays an important role in consumers’ purchase intention. Based on stimulus-organism-response theory, this paper further identifies social presence and flow experience as mediators and the consistency of Internet celebrities’ image and product image as a moderator and constructs an influence model of Internet celebrities’ interactivity on consumers’ purchase intention. The responses of a sample of 277 participants were collected by a questionnaire survey. SPSS and Amos were used to analyse the data. The results show that consumers’ social presence and flow experience mediate the positive impact of the interactivity of Internet celebrity anchors on influencing consumers’ purchase intention. However, there is no significant moderating effect of the consistency of Internet celebrities’ image and product image on the relationship between social presence or flow experience and purchase intention. A discussion and implications are offered.

## Introduction

With the maturity of live broadcasting technology and the development of online sales platforms, Internet celebrity livestreaming has become a novel sales mode. Through the live broadcasting platform, product information is presented more vividly in the form of trial and experience sharing in order to urge consumers to buy products (Lee and Overby, 2004). As a new marketing mode, Internet celebrity livestreaming not only provides consumers with good shopping experiences, but also brings a new profit model and cash channel to Internet celebrity anchors. Therefore, a practical question is worth further exploration: why do consumers purchase items in the live broadcasting room? In celebrity livestreaming marketing, the factors that affect consumers’ purchase intention have changed. The mechanism is also different from that in traditional e-commerce marketing. However, there is little research that notices this issue.

In psychology area, stimulation-organism-response (SOR) theory is often used to analyse the impact of external environment stimulation on an individual’s emotion and behaviour. Mehrabian and Russell (1974) believe that people are vulnerable to the stimulus and influence of external factors. When environmental factors (S) such as visual images and sounds are received by people, they will initially affect individuals’ internal state (O), and then psychological changes are generated, which further influence their behaviour (R). With the development of e-commerce, SOR theory has been widely applied in the field of online shopping. This study applies it to analyze a novel marketing phenomenon – online celebrity livestreaming. The intense interaction between celebrity anchors and consumers is a kind of stimulus to consumers, which will change their moods and inner states, and then ultimately influence purchase intention. This process is consistent with the SOR model.

The most significant feature of online live broadcasting is the real-time interaction between consumers and anchors (Fang, 2012). It may create novel consumer decision-making process different from that in traditional online shopping. Specifically, livestreaming anchors become shopping guides who introduce the product in detail by displaying the functions (Chen and Lin, 2018). The multichannel, real-time, dynamic and two-way interaction between anchors and consumers arouse consumers’ desire to purchase (Saffer et al., 2013; Liu et al., 2020). Thus, an anchor’s interactivity seems quite important in celebrity livestreaming marketing (Kelleher, 2009; Fang, 2012). The current study explores the mechanism by which the interactivity of Internet celebrity anchors affects consumer purchase intention.

In addition, live broadcasting can help consumers get access to information rapidly. A prominent feature of livestreaming marketing is that each consumer is not separate from other buyers but is at the presence of others (Greenleaf and Lehmann, 1995; Xie et al., 2016; Park et al., 2018). In offline retail stores, consumers perceive products through viewing, touching and direct interactions with salesmen. This effect also exists for online shopping, even more important than before. Social presence reflects the extent to which the anchor can produce a kind of intimacy to their audiences (Short et al., 1976). Anchors can create a subtle emotional experience for consumers through skilled interactions, which may enhance consumers’ sense of social preference (Shin and Shin, 2011). The audiences’ inner self can be aroused, which makes them completely immersed in live broadcasting and then stimulate their purchase intention for the products recommended.

Moreover, flow experience refers to the process that one acquires pleasure and well-being through participating in specific activities (Hoffman and Novak, 2009). In live broadcasting, anchors communicate with consumers through timely feedback or on-site displays, which may generate flow experience. This process helps audiences acquire product information and reduce risk perception. Consumers may also obtain a positive emotional experience of enjoyment and pleasure, which will promote their attachment and loyalty to the Internet celebrity anchor and then further stimulate their purchase intention. Chen and Lin (2018) examine the positive influence of flow experience livestreaming usage intention. The current research includes both social presence and flow experience in the consumer decision-making process model and examines their mediating roles in the relationship between interactivity of Internet celebrity anchors on consumer purchase intention.

Moreover, when celebrity image is consistent with product image, consumers will better trust the products that celebrities recommend. Thus, this paper introduces the variable of the consistency of Internet celebrity image and product image as a moderator in the model, which may explore the boundary of the prior proposed model.

In summary, this study has several contributions. Firstly, based on the SOR model, this study identifies the role of the interactivity of Internet celebrity anchors in Internet livestreaming marketing, which applies the model to explain new marketing phenomenon. Secondly, this paper explores the mediation effects of social presence and flow experience in the relationship between the interactivity of celebrity anchors and consumer purchase intention in order to help scholars and managers comprehensively understand the ‘black box’ when consumers make purchase decisions in live broadcasting. Thirdly, this paper tests the moderating role of consistency of Internet celebrities’ image and product image in consumer decision-making process, which helps both scholars and practitioners understand in what circumstances the model is more robust.

## Hypotheses Development

Previous studies propose that compared to traditional media, online activities provide a unique experience in interaction for the Internet users (Skadberg and Kimmel, 2004; Hassanein et al., 2009; Mollen and Wilson, 2010). This experience can help consumer easily perceive product information and build consumer trust and engagement toward sellers, and finally influence purchase intention (Wongkitrungrueng and Assarut, 2020). In live broadcasting, anchors introduce product information and respond to online consumers’ enquiries in a timely manner, which effectively urges consumers’ participation. Therefore, the interaction between anchors and consumers is the key factor that enhances purchase intention. Moreover, the interactivity between Internet celebrity anchors and consumers creates social presence, which reflects the feeling on being together in the virtual environment. Social presence shortens the psychological distance between anchors and online buyers. The high perceptions of social presence will lead to perceived satisfaction with the anchor, and then affect purchase intention. In another aspect, the fast and timely two-way interactions help consumers indulge themselves in live broadcasting – flow experience that often exists in online activities. Flow experience generates consumers’ attachment and loyalty to the anchors and enhances their willingness to buy the products recommended by the anchors (Ling et al., 2011).

## Interactivity of Internet Celebrity and Social Presence

Social presence refers to the degree to which an individual is regarded as a “real person” and the perceived degree of contact with others in the process of using media to communicate (Parker et al., 1976). Previous studies have focused on which social business technology factors and practices affect individual social presence, such as rich social information, virtual agents, three-dimensional displays, human-computer interactions, and remote presentations (Pavlou and Xue, 2007; Wang et al., 2016; Algharabat et al., 2018). The above research not only effectively explains the ability of communication media to transmit social signals but also demonstrates that computer media communications—such as chat boxes and online customer support centers—can become the media of social telepresence communication (Qiu and Benbasat, 2005). Social presence plays an important role in the online shopping environment, especially when there is a lack of face-to-face interaction between consumers and businesses.

In the context of e-commerce, it is difficult for consumers to feel the enthusiasm and warmth brought by face-to-face communication, which weakens consumers’ purchase intentions to a certain extent (Wang and Emurian, 2005). However, with the support of a variety of communication technologies, live broadcasting can quickly spread sound and images, and online consumers can send instant messages to interact with Internet celebrity anchors; thus, consumers will have a sense of being on the scene (Chen and Lin, 2018), which improves consumers’ perceptions of social interaction (Hu et al., 2017). Live broadcasting enables consumers to interact with the anchor and feel the active participation of other buyers, which can convey emotional elements in real life through scene construction, and anchors can provide consumers with a more authentic purchase experience through real expressions, actions and other body language and professional product demonstrations. This immersive experience is the sense of social presence. The real-time communication and the interaction between consumers and anchors can increase their familiarity with each other and shorten the psychological distance between them (Gunawardena and Zittle, 1997). This will help consumers feel the enthusiasm and existence of the anchors and in turn improve consumers’ social presence.

H1: The interactivity of Internet celebrity anchors is positively related to consumers’ social presence.

## Interactivity of Internet Celebrity and Flow Experience

Flow experience refers to the psychological state in which an individual is immersed in a certain activity (Ha et al., 2007). When individuals have flow experiences, they have a high sense of excitement and fulfillment, and they lose their perception of the passage of time and changes in the real world (Su et al., 2016). Concentration and enjoyment are two important parts of the immersion experience. Flow experiences not only entail personal concentration and enjoyment but also exploration and entertainment. That is, immersion experiences encourage consumers to participate in activities and have fun, and will continue to strengthen their participation due to the improvement of their subjective experiences (Skadberg and Kimmel, 2004; Lu and Wang, 2008).

With the development of the mobile Internet, flow experiences have been introduced into online consumer behavior research. Early studies have found that interactive technology enables more users to experience flow. When consumers are in a flow experience, the brand guides consumers in the interactive process and motivates consumers to create value for the brand, for example, spread brand reputation. (Hamilton et al., 2016). Effective social interaction can make consumers feel happy and in turn generate a flow experience (Animesh et al., 2011). In the process of live broadcasting, Internet celebrity anchors effectively convey product information. Consumers can interact with Internet celebrities through bullet screens, gifts, and other methods. When people perceive the interaction, they will participate in the interaction more actively, leading to a better emotional experience and the immersion state of flow (Gao et al., 2017). Therefore, interaction with Internet celebrity anchors improves consumer participation and immersion, causing consumers to forget the passage of time and enter a flow state.

H2: The interactivity of Internet celebrity anchors is positively related to consumer flow experience.

## Social Presence and Purchase Intention

Research on social presence is mostly based on the context of traditional web shopping platforms (Botha and Reyneke, 2016; Keng et al., 2016), and the live broadcast, a real-time “social situation,” is rarely taken into account in the interpretation of consumption behavior (Animesh et al., 2011). Consumers’ emotional responses to goods are the internal driving force of their purchase intentions. Social presence makes consumers experience positive emotions, such as pleasure, which positively affects consumer attitudes and loyalty (Hassanein and Herd, 2007). Positive emotion can establish a bond between consumers and commodities, stimulate consumers to actively participate in commodity marketing, help consumers develop a sense of social presence in the virtual network, and finally form consumers’ purchase intentions.

In livestreaming marketing, the continuous, in-depth interactions between the anchor and the consumer can improve consumers’ sense of social presence, inspire positive emotions in consumers, and reduce consumers’ uncertainties and concerns about products, thus improving their willingness to purchase (Dawes and Nenycz-Thiel, 2014; Li et al., 2020). At the same time, pleasant emotions improve the perceived alignment between products and consumer needs, shorten the psychological distance between consumers and online merchants, and promote consumer purchase intention (Gefen and Straub, 2004; Gao et al., 2018). Therefore, this paper puts forward the following hypothesis:

H3: Social presence is positively related to purchase intention.

## Flow Experience and Purchase Intention

Flow experience can affect consumers’ purchase intentions *via* pleasure and satisfaction. When consumers are in the state of flow, they have a great sense of pleasure and psychological satisfaction, and these positive emotional reactions may promote purchase behavior (Hausman and Siekpe, 2009; Kim et al., 2017). When online consumers are in the state of flow experience, they develop purchase intentions to extend the positive emotions associated with the flow state (Animesh et al., 2011). For example, consumer flow experiences on brand websites promote positive emotions and in turn inspire positive effects, such as increased brand loyalty, which can then be transformed into purchasing behaviors (Shim et al., 2015).

In live broadcast marketing, the live broadcast platform provides consumers with a panoramic interactive experience of vision and hearing and creates a realistic shopping experience. This immersive experience can stimulate consumers’ desire to buy. Moreover, the real-time interaction, natural language, and personalized attention of network media contribute to consumers’ flow experiences. The flow experience immerses consumers in the communications of the Internet celebrity anchor, encouraging them to ignore surrounding visual information that is irrelevant to the live broadcast and promoting their understanding and acceptance of the information conveyed by the Internet celebrity anchor (Webster et al., 1993; van Noort et al., 2012). Therefore, this paper puts forward the following hypothesis:

H4: Flow experience is positively related to purchase intention.

## The Mediating Roles of Social Presence and Flow Experience

Stimulation-organism-response theory is widely used in online shopping research. This theoretical model proposes that the stimulation of environmental factors ultimately affects people’s reactions by influencing their internal psychological states (Akerlof, 1970). The stimulus is the factor that promotes individual behavior in the external environment. The organism is a psychological transformation mechanism by which individuals internalize stimulating factors into information as the basis for the final behavior. The response is the final psychological or behavioral reaction of the stimulated object. For example, Eroglu et al. (2003) applies SOR theory to the research in the field of online shopping, the atmosphere cues of shopping websites are operationalized as the “stimulus,” the user’s internal emotional state (such as perceived pleasure, arousal) as the “organism” and user satisfaction and approach or avoidance behaviours as the “response,” thus a research model of online shopping atmosphere was constructed, which proposed that the atmospheric cues of the online store influence shoppers’ emotional and cognitive states, which then affect their shopping outcomes.

In Internet celebrity livestreaming marketing, the interaction between the anchor and consumers in the live broadcast room is an external stimulus to consumers that promotes positive emotions. Continuous, frequent interactions allow consumers to experience a high level of social presence and a strong flow state. The resulting positive emotions encourage consumers to have positive attitudes toward Internet celebrities and products. At the same time, a good immersion experience can promote information processing and thus improve consumer memory of product information (Webster et al., 1993; van Noort et al., 2012). For example, in TV programs, the higher the audience’s investment level, the easier it is for the audience to recall the brands implanted in the program (Balasubramanian et al., 2006). Therefore, based on SOR theory, positive interaction promotes consumer social presence and flow experiences, which may improve consumers’ acceptance of product information and finally stimulate consumer purchase intention. Therefore, this paper puts forward the following hypotheses:

H5: Social presence plays a mediating role in the relationship between the interactivity of Internet celebrity anchors and purchase intention.

H6: Flow experience plays a mediating role in the relationship between the interactivity of Internet celebrity anchors and purchase intention.

## The Moderating Role of the Consistency Between Internet Celebrity Image and Product Image

Consistency refers to the consistency of celebrity image and product at first, that is, the matching degree between celebrity and its products (Friedma, 1970), which is one of the decisive factors in the success of celebrity endorsement marketing. This consistency has a significant impact on the effects of advertising and consumer evaluation of products. When consumers perceive consistency between the celebrity image and the product image, they have more positive attitudes toward the celebrity image, find the celebrity more attractive and credible, and exhibit higher recognition and purchase willingness toward the products and brands the celebrity endorses (Jaideep et al., 1997). This study defines the consistency of Internet celebrity image and product image as follows: the degree of consistency between the image of Internet celebrity anchor and the image of products bought and sold in the live broadcast room (Liu et al., 2020).

The matching of Internet celebrities’ image with products affects consumer psychology and behavior (Liu et al., 2020). When the consistency between an Internet celebrity image and a product image is high, consumers will consider that online celebrities have high attraction and credibility (Friedma, 1970; Koernig and Boyd, 2009), which improves consumer willingness to purchase. In this situation, the experiences of social presence and flow also stimulate consumer purchase intention. On the contrary, when the consistency between the Internet celebrity image and the product image is low, it is difficult for consumers to establish trust in online celebrities and products, and the perception of product uncertainty increases, which weakens purchase intention. Furthermore, the effects of social presence and flow experience on consumers’ purchase intentions are also weakened. Therefore, this paper puts forward the following hypotheses:

H7: The consistency between Internet celebrity image and product image plays a moderating role in the effect of social presence on purchase intention, such that the effect is stronger when the consistency between Internet celebrity image and product image is low.

H8: The consistency between Internet celebrity image and product image plays a moderating role in the effect of flow experience on purchase intention, such that the effect is stronger when the consistency between Internet celebrity image and product image is low.

## Materials and Methods

This study used a questionnaire to test the hypothesised model. The questionnaires were posted *via* Sojump,^1^ a large-scale online survey platform in China that is widely used in behavioural and psychological research (Li et al., 2018). We distributed the survey link in a popular livestreaming shopping forum. The respondents were the groups who made livestreaming purchases recently. Participant were asked to recall his or her most recent livestreaming shopping experience before they completed the survey. Finally, 277 valid questionnaires were obtained, with an effective recovery rate of 92.3%. The sample size met Hair et al.’s (2009) recommendation. In terms of the demographic information, the proportions of men and women are balanced, with 58.1% men and 41.9% women. The majority of the respondents earn more than RMB 4000 a month (63.1%) and have a bachelor’s degree (63.9%), while 53.4% of the respondents engage in Internet live broadcast shopping one to three times a month.

## Measures

To ensure the reliability and validity of the questionnaire, the scales in this study are all derived from the established scales in the existing studies, and the scales are adapted according to the purpose of this study. Gender, age, education, occupation, income and purchase frequency in the live broadcasting room are included as the control variables. Interactivity (Cronbach’s α=0.743) and the consistency of Internet celebrities’ image and product image (Cronbach’s α=0.803) are measured by three items, respectively, from Liu et al. (2020). Sample items are ‘The celebrity anchor has good interactions with the audiences’ and ‘The celebrity image is well matched with the product image he/she recommends’. In addition, social presence (Cronbach’s *α* =0.779) and flow experience (Cronbach’s *α* =0.747) are measured by three items adapted from Hassanein et al. (2009) and Huang et al. (2017), respectively. Sample items are ‘There is a sense of human contact in this livestreaming room’ and ‘I am experiencing flow in this livestreaming room’. Lastly, three items from Dodds et al. (1991) were used to measure purchase intention (Cronbach’s *α* =0.779). A sample item is ‘The likelihood of purchasing this product is: (very high to very low)’. All the constructs are measured with five-point Likert scales.

## Results

### Reliability and Validity

To ensure construct validity for each variable, exploratory factor analysis was conducted followed by the calculations of AVE and CR to assess the convergent validity of the measurement model. The factor loadings of each indicator exceed the accepted value of 0.5. The values of AVE of all the constructs exceed 0.5, indicating that the scales have good convergent validity (Fornell and Larcker, 1981). All the items’ composite reliability values are above the benchmark of 0.60, showing that the measurement model has good internal consistency. Furthermore, Table 1 reveals that the square root of AVE for each latent variable is greater than its correlation coefficient with other latent variables, which shows good discriminant validity (Barclay et al., 1995). The reliability coefficients for all the constructs are greater than 0.7 and are accepted based on George and Mallery’s (2003) criterion. These results provide a basis for further analysis.

**Table 1:** Descriptive statistics and correlation coefficients and discriminant validity model.

Variable	1	2	3	4	5
1. Interactivity	0.716				
2. Social presence	0.680^**^	0.766			
3. Purchase intention	0.586^**^	0.636^**^	0.741		
4. The consistency of Internet celebrities’ image and product image	0.656^**^	0.556^**^	0.565^**^	0.759	
5. Flow experience	0.681^**^	0.655^**^	0.625^**^	0.589^**^	0.715

*N=277*.

** *p<0.01. The value on the diagonal represents the square root of the AVE value*.

### Hypothesis Testing

Amos 24.0 was used to test the path coefficient and hypothesis. The results (*χ*^2^/df=2.679, RMSEA=0.078, GFI=0.908, IFI=0.934, TLI=0.912, CFI=0.933) suggest that the theoretical model fits the data quite well according to the thresholds (Fornell and Larcker, 1981; Marsh et al., 1988; Kline, 2011).

**Figure 1:**
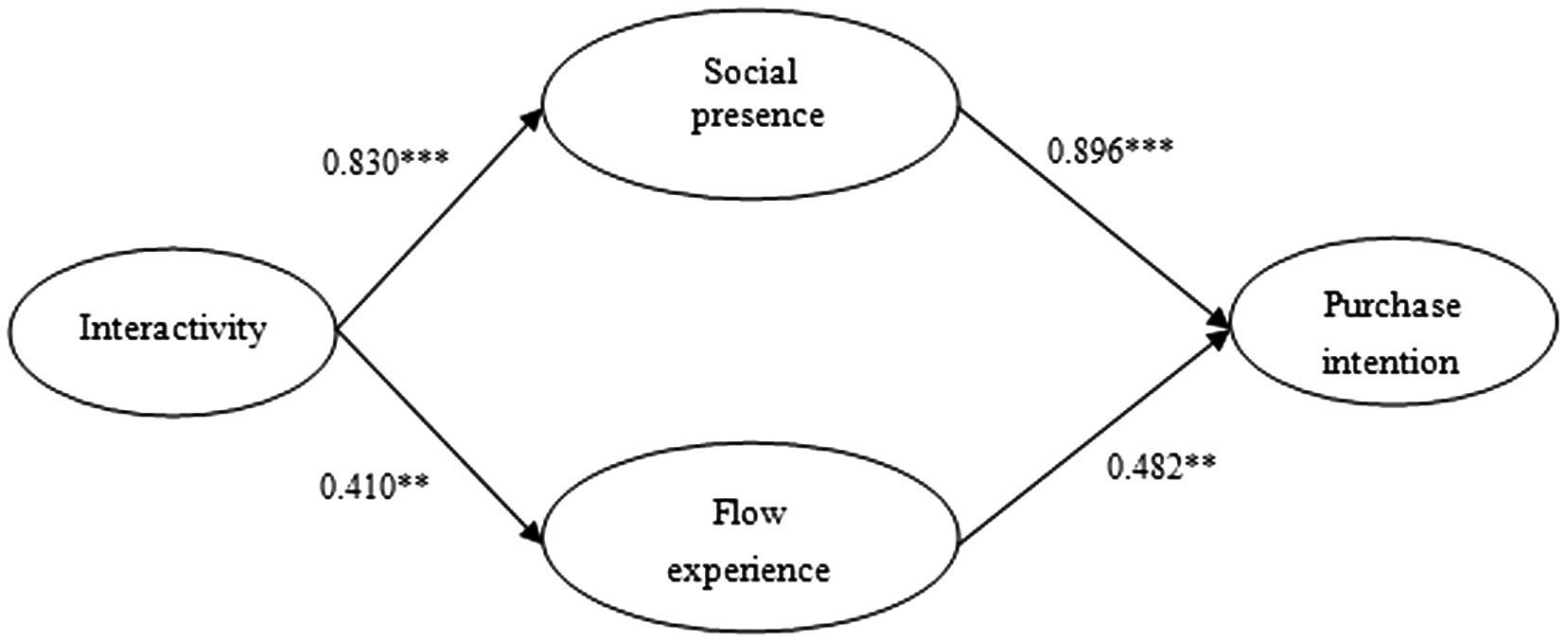
Path coefficients of the hypothesized model.^**^p<0.01, ^***^p<0.001.

Figure 1 shows the results of each standardized path coefficient in the model. Specifically, interactivity has significant positive impacts on both social presence and flow experience (*β* =0.830, *p* <0.001; *β* =0.410, *p* <0.01), adequately supporting Hypothesis 1 and Hypothesis 2. Meanwhile, both social presence and flow experience exert positive impacts on purchase intention (*β* =0.896, *p* <0.001; *β* =0.482, *p* <0.01). Hypothesis 3 and Hypothesis 4 are supported.

To examine mediation effect, we adopted the bootstrap method proposed by Hayes (2013). The results are shown in Table 2. LLCI and ULCI are the lower and upper limits for the 95% confidence intervals, respectively, and the 95% CI does not contain 0. The results show that the mediating effects of social presence and flow experience on the relationship between interaction quality and purchase intention are significant. Thus, Hypothesis 5 and Hypothesis 6 are supported.

**Table 2:** Mediating effect test.

Effect	Estimated value	*P*	Standard Error	LLCI	ULCI
Interactivity → Social presence → Purchase intention	0.340	0.010	0.126	0.163	0.574
Interactivity → Flow experience → Purchase intention	0.431	0.004	0.141	0.215	0.673

To further test the moderating effect, we conducted a series of hierarchical regression analyses with SPSS 25.0 (Hox, 2010). In order to verify the interactions between independent variables and moderating variables, we first centralized all the variables and calculated the interaction terms (Aiken and West, 1991). The regression results are shown in Table 3. In models 4 and 8, the interaction items have no significant effect on purchase intention (*β*= −0.024, *p>* 0.05; *β*= −0.059, *p>* 0.05). Thus, Hypothesis 7 and Hypothesis 8 are not supported.

**Table 3:** Results of the moderated regression analyses.

Variables	Purchase intention						
	Model 1	Model 2	Model 3	Model 4	Model 5	Model 6	Model 7
Age	−0.062	−0.060	−0.066	−0.067	−0.045	−0.054	−0.056
Gender	0.012	−0.022	0.000	−0.001	0.005	0.020	0.022
Education	0.129^*^	0.091	0.075	0.072	0.106^*^	0.087	0.078
Occupation	0.059	−0.026	−0.022	−0.019	0.019	0.013	0.017
Income	0.020	0.064	0.079	0.080	0.041	0.060	0.061
Purchase frequency	−0.331^***^	−0.168^***^	−0.127^**^	−0.126^**^	−0.180^***^	−0.141^**^	−0.136^**^
Social presence		0.599^***^	0.451^***^	0.451^***^			
Flow experience					0.580^***^	0.428^***^	0.426^***^
The consistency of Internet celebrities’ image and product image			0.285^***^	0.290^***^		0.278^***^	0.293^***^
Social presence × The consistency of Internet celebrities’ image and product image				−0.024			
Flow experience × The consistency of Internet celebrities’ image and product image							−0.059
*R* ^2^	0.096	0.424	0.477	0.475	0.412	0.459	0.461
*∆R* ^2^	0.115^***^	0.323^***^	0.054^***^	0.001	0.311^***^	0.048^***^	0.003
*F*	5.874	29.985	32.436	28.786	28.631	30.318	27.203

*N=277*.

* *p<0.05*,

** *p<0.01*,

*** *p<0.001*.

## Discussion

Based on SOR theory, this study uses a situational questionnaire to collect data and examines the relationship between the interactivity of Internet celebrities and purchase intention. The conclusions are as follows: (1) the interactivity of Internet celebrities has a significant positive impact on consumer purchase intention; (2) social presence and flow experience mediate the impact of the interactivity of Internet celebrities on consumers’ purchase intention; and (3) the consistency of Internet celebrities’ image and product image has no moderating effect on the relationship between social presence or flow experience and consumer purchase intention. The reasons may be as follows: First, with the rapid development of the Internet celebrity economy, the types of goods recommended by Internet celebrities have become diversified and are no longer limited to a certain type of product. Take Mr. Li Jiaqi, the “No. 1 Best Livestreaming Seller in China” as an example: although he was originally famous for selling cosmetics, currently, the categories of products in his live broadcasting room range from food to daily necessities.

Therefore, when consumers buy products in livestreaming rooms, they no longer focus on the matching degree between the Internet celebrities’ image and the product image but are more probably based on their trust on Internet celebrities. Second, with the prevalence of livestreaming marketing, most of the ‘performance’ is similar, which makes consumers inevitably have “aesthetic fatigue.” They are more likely to buy multiple categories of products in their favourite live broadcasting rooms. Therefore, the consistency of Internet celebrities’ image and product image on consumers is not that important.

## Theoretical Implications

This research has the following theoretical contributions. First, theoretical research on Internet livestreaming marketing obviously lags behind practical development. The feature of livestreaming marketing is the two-way, real-time interaction between Internet celebrities and fans (Haimson and Tang, 2017). The exploration on how to make use of this advantage to stimulate purchase intention is scarce in the existing literature. Based on the SOR model, this study identifies the interactivity of Internet celebrities as an independent variable and examines its influence on consumer purchase intention in livestreaming marketing.

Second, unlike the traditional face-to-face marketing model, it is believed that livestreaming marketing lacks a ‘human touch’. To some extent, the shortage of effective social interaction weakens consumers’ purchase intention (Wang and Emurian, 2005). How to improve consumers’ perception of social interaction in online shopping is an important issue. This study identifies social presence and flow experience as the mediators and tests the mediation effect, which can help scholars and managers understand the ‘black box’ in livestreaming marketing.

Third, this paper finds the consistency of Internet celebrities’ image and product image has no moderating effect on the relationships between social presence or flow experience and purchase intention, which is inconsistent with the conclusion in some previous research (e.g., Liu et al., 2020). A possible reason is that with the rapid development of Internet celebrity economy, a celebrity is no longer limited to specific categories of commodities. In order to save time, consumers tend to buy most of the products they need from a few familiar anchors.

## Practical Implications

First, the Internet celebrity anchor should pay attention to the interaction with consumers in live broadcasting. Consumers’ shopping experience is extremely important. A high-quality live interaction can effectively promote the participation of consumers so that consumers will have a positive emotional perception of the anchor, resulting in purchase intention. The key to live interaction is to mobilize consumers’ enthusiasm. Internet celebrities should pay attention to language skills. They must actively provide timely feedback to consumers’ problems, encourage consumers to participate in interactions and create a warm and pleasant atmosphere for consumers.

Second, the integration of social presence and flow experience can enhance purchase intention. In live broadcasting, consumers are encouraged to express their views or experiences related to the products. The social presence transfers personal consumption behaviour into social consumption behaviour. In addition, real-time interaction makes consumers not just an information receiver, but also the main part in live broadcasting. This process provides consumers with emotional belongingness and value identification (Pozharliev et al., 2017), which creates a flow experience for consumers to stimulate their purchase intention. The application of advanced technology, such as 5G and VR technology, will bring better sensory experience to consumers in the near future.

## Limitations and Future Research

There are some limitations in the current research. Firstly, this paper explores the influence of Internet celebrity interactivity on purchase intention without considering consumer traits or product types. In the future study, these factors should be included to depict a more comprehensive decision-making process. Secondly, the sample is not large enough. In the future scholars should replicate the study with a larger sample to further validate the findings. Finally, this paper used a questionnaire survey to collect data. In the future, the experimental method can be used to examine the causal relationship between the variables. The findings generated with different research methods can provide deeper insights in this area.

## Data Availability Statement

The raw data supporting the conclusions of this article will be made available by the authors, without undue reservation.

## Ethics Statement

Ethical review and approval was not required for the study on human participants in accordance with the local legislation and institutional requirements. Written informed consent for participation was not required for this study in accordance with the national legislation and the institutional requirements.

## Author Contributions

WS and WG worked on the theory development, literature review, and paper writing. RG worked on paper writing, data collection, and analysis. All authors contributed to the manuscript and approved the submitted version.

## Conflict of Interest

The authors declare that the research was conducted in the absence of any commercial or financial relationships that could be construed as a potential conflict of interest.

## Publisher’s Note

All claims expressed in this article are solely those of the authors and do not necessarily represent those of their affiliated organizations, or those of the publisher, the editors and the reviewers. Any product that may be evaluated in this article, or claim that may be made by its manufacturer, is not guaranteed or endorsed by the publisher.

## Footnotes

^1^ http://www.sojump.com
